# DeDaL: Cytoscape 3 app for producing and morphing data-driven and structure-driven network layouts

**DOI:** 10.1186/s12918-015-0189-4

**Published:** 2015-08-14

**Authors:** Urszula Czerwinska, Laurence Calzone, Emmanuel Barillot, Andrei Zinovyev

**Affiliations:** Institut Curie, 26 rue d’Ulm, Paris, France; INSERM U900, Paris, France; Mines Paris Tech, Fontainebleau, France

## Abstract

**Background:**

Visualization and analysis of molecular profiling data together with biological networks are able to provide new mechanistic insights into biological functions. Currently, it is possible to visualize high-throughput data on top of pre-defined network layouts, but they are not always adapted to a given data analysis task. A network layout based simultaneously on the network structure and the associated multidimensional data might be advantageous for data visualization and analysis in some cases.

**Results:**

We developed a Cytoscape app, which allows constructing biological network layouts based on the data from molecular profiles imported as values of node attributes. DeDaL is a Cytoscape 3 app, which uses linear and non-linear algorithms of dimension reduction to produce data-driven network layouts based on multidimensional data (typically gene expression). DeDaL implements several data pre-processing and layout post-processing steps such as continuous morphing between two arbitrary network layouts and aligning one network layout with respect to another one by rotating and mirroring. The combination of all these functionalities facilitates the creation of insightful network layouts representing both structural network features and correlation patterns in multivariate data. We demonstrate the added value of applying DeDaL in several practical applications, including an example of a large protein-protein interaction network.

**Conclusions:**

DeDaL is a convenient tool for applying data dimensionality reduction methods and for designing insightful data displays based on data-driven layouts of biological networks, built within Cytoscape environment. DeDaL is freely available for downloading at http://bioinfo-out.curie.fr/projects/dedal/.

## Background

One of the major challenges in systems biology is to combine in a meaningful way the large corpus of molecular biology knowledge recapitulated in the form of large interaction networks together with high-throughput omics data [1].

There exist numerous methods using biological networks for making insightful high-throughput data analysis [1]. These methods can be separated in three large groups, concentrating on: (1) mapping the data on top of a pre-defined biological network layout, (2) identifying subnetworks from a global network possessing certain properties computed from the data (such as subnetworks enriched with differentially expressed genes), and (3) using biological network structure for pre-processing the high-throughput data (for example, for “smoothing” the discrete mutation data).

Quantitative omics data can be mapped on top of a pre-defined biological network layout. Currently, most of the pathway databases (such as KEGG [2], Reactome [3]) already provide these features using simple and advanced data visualization tools. Omics data visualization tools onto networks are constantly improving and become more elaborated [4]. For example, the VANTED tool [5] creates a classification tree according to the KEGG pathway hierarchy and shows a biological network with omics data as barplots or pie-charts attached to the nodes allowing the visualization of complex data with other means than simple node coloring. NaviCell [6, 7] and related pathway database Atlas of Cancer Signalling Network (ACSN) together with standard heat maps and barplots provide more flexible data visualization tools such as glyphs (symbols with configurable shape, size and color) and map staining (using the network background for visualization) [8, 9]. An interesting approach for data visualization using biological networks was developed in NetGestalt online tool [10]. This tool is based on a NetSAM R package to create modules by hierarchical ordering of the network in one dimension and visualizes high-throughput data according to a chosen track as a combination of barplots and heat maps.

Omics data are used to identify overexpressed or enriched subnetworks. For example, in [11], expression data were combined with network information in order to identify under- or overexpressed subnetworks in Huntington’s disease and breast cancer. Inspired by this method, several Cytoscape plug-ins were developed and applied to various omics data in order to find connected sub-components where most of the genes were differentially expressed or co-expressed [12, 13]. A recent review presents the integration of molecular profiles with networks in order to find “network modules” [14].

Projection of the high-throughput data into the basis of smooth functions defined on a biological network graph was suggested in [15]. Recently, biological networks were used to regularize the genome-wide mutational landscapes (which are sparse) in cancer, applying network smoothing methods [16].

However, none of the methods cited above had the purpose to visualize high-throughput data by computing a specific network layout based on the omics data themselves, which would combine both the network structure and the data associated to the network node attributes. Some of the existing Cytoscape layout algorithms (such as Group Attributes Layout) allow exploiting the values of single node attributes, but this possibility is currently under-developed. We believe that using networks for visualizing and analyzing data requires methods that would be able to create more suitable biological network layouts adapted for a particular task.

Mathematically speaking, molecular entities exist in two metric spaces. The first one is the space of biological functions, where the distance between two molecules can be defined by the number of steps (edges) in a graph defining pairwise functional relations (such as protein-protein interactions) along the shortest path connecting them. The other metric space is the data space, where the distance between two molecules is defined by the proximity of the corresponding numerical descriptors (such as expression profiles). The network distances are usually visualized by designing a 2D or 3D layout, representing the network structure. Visualization of distances in data space is achieved by data dimension reduction methods (such as PCA) projecting multidimensional vectors in 2D or 3D space.

Dimension reduction techniques were already used for producing biological network layouts. For example, GOlorize Cytoscape plugin computes the layout of a biological network using the results of GO enrichment analysis [17]. Some existing methods apply multidimensional scaling to the distance matrix defined by the graph path distance [18]. These methods have the purpose to construct more appealing network layouts. However, they usually do not use the multidimensional data attached to the network nodes. Therefore, they can not be adapted for visualization of a particular dataset. On the other hand, there exist a number of convenient software allowing the construction and visualization of the structure of correlation graphs, computed from multidimensional data, e.g., BioLayout Express3D [19] or Arena3D [20]. However, these representations do not use the knowledge of the structure of real biological networks since they are not inferred from the data by correlation analysis.

We believe that in certain analyses, it could be insightful to construct the biological network layout based simultaneously on the network structure and the associated multidimensional data. One possible solution consists in applying data dimension reduction techniques. In order to allow Cytoscape users to conveniently apply linear and non-linear dimension reduction methods accompanied by network-based data regularization, we have developed DeDaL, a Cytoscape 3 app for computing and mixing data-driven and structure-driven network layouts. Unlike many other methods, the purpose of DeDaL is not to improve the visual appeal of the biological network layout, but to modify it in such a way that the trends in the associated data and exceptions from these trends would be detectable more easily.

## Implementation

DeDaL is a *simplified* Cytoscape 3 app implemented in Java language. For computing linear and non-linear principal manifolds, DeDaL uses VDAOEngine Java library (http://bioinfo-out.curie.fr/projects/elmap/). For computing the eigenvectors of a symmetric Laplacian matrix, the parallelized Colt library has been used (http://acs.lbl.gov/ACSSoftware/colt/). Internal graph implementation is re-used from BiNoM Cytoscape plugin [21–23]. The source code of DeDaL is available at http://bioinfo-out.curie.fr/projects/dedal/.

### Producing data-driven network layouts

Data-driven network layout (DDL) is produced by DeDaL by positioning the nodes of the network according to their projection from the multidimensional data space of associated numerical vectors into a 2D space. DeDaL implements three algorithms for performing this dimension reduction: (1) projection onto a plane of two selected principal components; (2) projection onto a non-linear 2D surface approximating the multidimensional data distribution, i.e. principal manifold, computed by the method of elastic maps [24–28]; and (3) use of (1) or (2) preceded by network-based regularization (smoothing) of the data, based on computing the *k* first eigenvectors of the Laplacian matrix of the network graph and projecting data into this subspace (as suggested in [15]).

DeDaL implements specific data pre-processing and resulting layout post-processing steps. Pre-processing steps include (1) selecting only nodes whose associated numerical vectors (imported as tables into Cytoscape) are sufficiently complete and (2) optional double centering of the data matrix. Post-processing of the resulting layout includes (1) avoiding overlap between node positions by moving them in a random direction at a small distance; (2) moving the outliers (nodes positioned too distantly from other nodes) closer to the barycenter of the data distribution; and (3) placing the nodes with missing data into the mean point of the position of their network neighbours.

In future work, an effort will be made to project the data in the three dimensional space, or exploit the concept of multi-level 2.5D network representation [29]. We would like to let the user rotate the network layout in order to better visualize the network substructures which are difficult to represent in 2D space, as it is done in BioLayout 3D software [19]. We also plan to implement in DeDaL more flexible dimension reduction algorithms such as multidimensional scaling which will extend the data representation possibilities, better answering to specific user’s needs (for example, by using non-Euclidean metrics for comparing the molecular profiles). Finally, more sophisticated strategies of network layout morphing will be developed, taking into account the data. We will also improve the function for avoiding extensive node overlapping.

### Manipulating network layouts in DeDaL

In order to allow the comparison of the resulting DDLs with standard layouts produced by Cytoscape and to transform one into another, DeDaL implements simple layout morphing and aligning methods. Morphing of two network layouts is performed by a linear transformation, moving matched nodes along straight lines. DeDaL provides a convenient user dialog for morphing one layout into another so that the user can immediately appreciate the morphing result. The morphing operation provides poor results if one layout is systematically rotated or flipped with respect to the node positions in another one. DeDaL allows aligning two network layouts by rotating, mirroring, and minimizing the Euclidean distance between two layouts.

### Double-centering the data matrix

The data matrix is optionally double-centered by subtracting from each matrix entry the mean value calculated over the corresponding matrix row and the mean value calculated over the matrix column, followed by adding the global mean value computed over all matrix entries. This procedure eliminates some global biases in the data such as the global differences in average fluorescence intensity of different probes in microarray data.

### Network-based smoothing of data

DeDal perfoms Network data smoothing, as suggested in [15]. For a graph representing the biological network, its Laplacian and all its eigenvectors are computed. These vectors define a new orthonormal basis in the multidimensional data space. To smooth the values of the data matrix, the initial multidimensional vector associated to a datapoint is projected into the subspace spanned by the first smallest *k* eigenvectors of the graph’s Laplacian. DeDaL smoothing parameter is defined by $p_{\textit {ns}}=1-\frac {k-(n_{c}+2)}{N-(n_{c}+2)}, p_{\textit {ns}}\in \, [0;1]$, where *n*_*c*_ is the number of connected components in the graph and *N* is the number of nodes on the graph. Therefore, *p*_*ns*_=0 corresponds to *k*=*N*, i.e. when no smoothing is performed and all eigenvectors are used, while *p*_*ns*_=1 corresponds to *k*=(*n*_*c*_+2) and the first two non-degenerated eigenvectors are used to smooth the data. In the latter case, the data become effectively three-dimensional, with the first dimension corresponding to the average value of the data matrix computed over each connected component of the graph.

### Exporting the pre-processed data

The results of pre-processing the data for a given network can be exported to a file. Actually, two files are created: one in a simple tab-delimited format suitable for further analyses in most of statistical software packages and another file in the “.dat” format, suitable for analyses in ViDaExpert multidimensional data visualization tool [30]. That way, network smoothing of an expression dataset can be done for further application in any machine learning algorithms (clustering, classification). For this purpose, DeDaL can be also used in a command line mode (see examples on the website, http://bioinfo-out.curie.fr/projects/dedal/).

### Computing principal components and principal manifolds

The principal components in DeDaL are computed using singular value decomposition by the method that allows the treatment of missing data values without pre-imputing them, as it is described in [31]. Data points, containing more than 20 % of missing values are filtered out from the analysis. DeDaL computes the 10 first principal components if there are more than 10 data points, and *k* principal components if there are *k*+1 data points, with *k*<10. After computing the principal components, DeDaL reports the amount of variance explained by each of the principal components.

Time-efficient method of elastic maps for computing principal manifolds [24–28] also allows dealing with missing data without pre-imputing them. In this case, a 2D rectangular manifold is computed, and the amount of variance explained by it is reported.

### Continuous layout morphing

Morphing two network layouts is performed by a simple linear transformation. A node having position (*x*_11_,*x*_12_) in the initial layout and the position (*x*_21_,*x*_22_) in the target layout is placed during the morphing procedure in the position (*p*×*x*_21_+(1−*p*)*x*_11_,*p*×*x*_22_+(1−*p*)*x*_12_), where *p*∈ [0;1] is the morphing parameter representing the fraction of distance between the initial and target node positions along the straight line.

### Aligning two network layouts by rotation and mirroring

Morphing between two network layouts might be meaningless if all nodes in one layout are systematically rotated or flipped with respect to the node positions in another layout. This situation is often the case when producing the pure data-driven layout and comparing it to the initial structure-driven layout. In this case, DeDaL allows minimizing the Euclidean distance between two layouts defined as the sum of squared Euclidean distances between all matched nodes with respect to all possible rotations and mirroring of one of the layouts. DeDaL provides an option to align networks before morphing them. Also, a user can align several network layouts to one chosen reference network layout, using a separate “Layout aligning” dialog. For example, it is usually useful to align the structure-driven layouts to the PCA-based data-driven layout.

### Using DeDaL in command line mode

DeDaL can be used separately from the Cytoscape environment, in the command line mode, as it is explained on the DeDaL website with several examples. This is especially recommended for computing data-driven layouts for large networks containing more than ten thousand nodes. Command line mode allows applying all data pre-processing steps, including double-centering and network smoothing, saving the resulting network layout as a XGMML file and saving the eigenvector decomposition of the Laplacian of the network graph for future use.

## Results

### Using TCGA transcriptome data and HPRD network

We used The Cancer Genome Atlas (TCGA) transcriptomic dataset for breast cancer (548 patients)[32] and Human Protein Reference Database (HPRD) database [33] as a source of protein-protein interaction network.

**Fig. 1:**
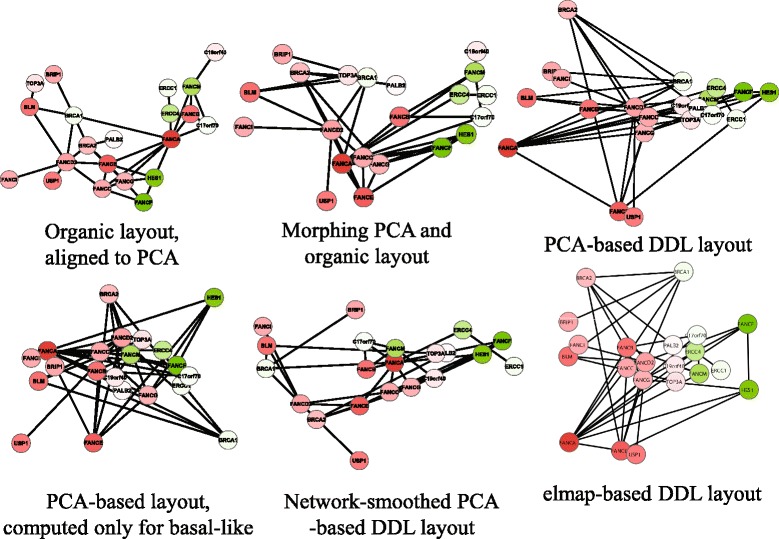
Using DeDaL for visualizing Fanconi pathway in breast cancer. Top row: standard organic layout (left), PCA-based DDL (right), morphing two previous layouts at half-distance (middle). Bottom row from left to right: PCA-based DDL computed only for basal-like tumors (note change in position of BRCA1 gene), PCA applied to network-smoothed profile, DDL computed using elastic map (elmap) algorithm for computing non-linear principal manifold. Red color denotes larger positive values of the t-test computed for the difference between basal-like and non basal-like groups of samples, green color corresponds to negative values of the t-test

Firstly, as an example of a small subnetwork, we selected proteins involved in Fanconi DNA repair pathway [34] as it is defined in Atlas of Cancer Signaling Network [8, 9]. For node coloring, we mapped the value of the t-test computed for the gene expression difference between the basal-like (one of the molecular subtypes of breast cancer, significantly contributing to the intertumoral variability) and non basal-like breast tumors. We have imported the TCGA data in Cytoscape and applied DeDaL for the transcription levels of the genes in the subnetwork (Fig. 1).

One can see (Fig. 1, top right) that the first principal component sorts the nodes according to the t-test, because in this case the first principal component is associated with the basal-like breast cancer subtype. The second principal component gives additional information such as the fact that the expression levels of BRCA2 and FANCE are differently modulated, though both are upregulated in the basal-like subtype. Morphing the organic network layout (Fig. 1, top left) with the PCA-based layout moves the position of some of the genes, keeping the general pattern of PCA preserved, while better reflecting the network structure (Fig. 1, top middle).

We have also applied PCA-based DDL to the subset of basal-like breast tumors (Fig. 1, bottom left) which showed the specific role of BRCA1 gene in this subtype confirming a known fact. Also, the position of USP1 gene has significantly changed with respect to the PCA-based DDL produced for the whole set of samples. This demonstrates the ability of DeDaL to produce network layouts specific for a particular cancer subtype.

Application of network smoothing is demonstrated in Fig. 1, bottom middle. The layout preserves the general pattern of the PCA-based DDL, while better visualizing the network structure, and moving some proteins into a different position. For example, BRCA1 gene is moved to the left because it is connected to several genes overexpressed in basal-like breast cancer subtype. Figure 1, bottom right, shows the application of non-linear PCA to data dimension reduction. This network layout better resolves the relations between some gene expression levels such as FANCF and HES1 and the roles of BRCA1 and BRCA2 in Fanconi DNA repair pathway.

### Visualizing RNA-Seq tissue expression data onto the network of tissue-specific genes

In order to illustrate the added value of DeDaL for visualizing expression data on top of relatively large networks, we constructed a tissue-specific subnetwork from HPRD global network of protein-protein interactions (PPIs) using the following approach. RNA-Seq data containing transcriptomes for 27 healthy human tissues were obtained from [35]. Replicate measurements were averaged in order to obtain a single transcriptomic profile per tissue. In each profile, the genes were ranked according to their expression and the most significant largest connected component (LCC) of the global PPI network directly connecting the top ranked genes (OFTEN subnetwork) was identified using BiNoM plugin [21, 22] (see detailed methodology description in [36]). After this step, the tissue-specific subnetworks that showed a significant score for the size of LCC were merged. This resulted in a network containing 1047 nodes, representing the top genes that are highly expressed in at least one tissue type, and 1986 edges representing direct PPIs between them.

**Fig. 2:**
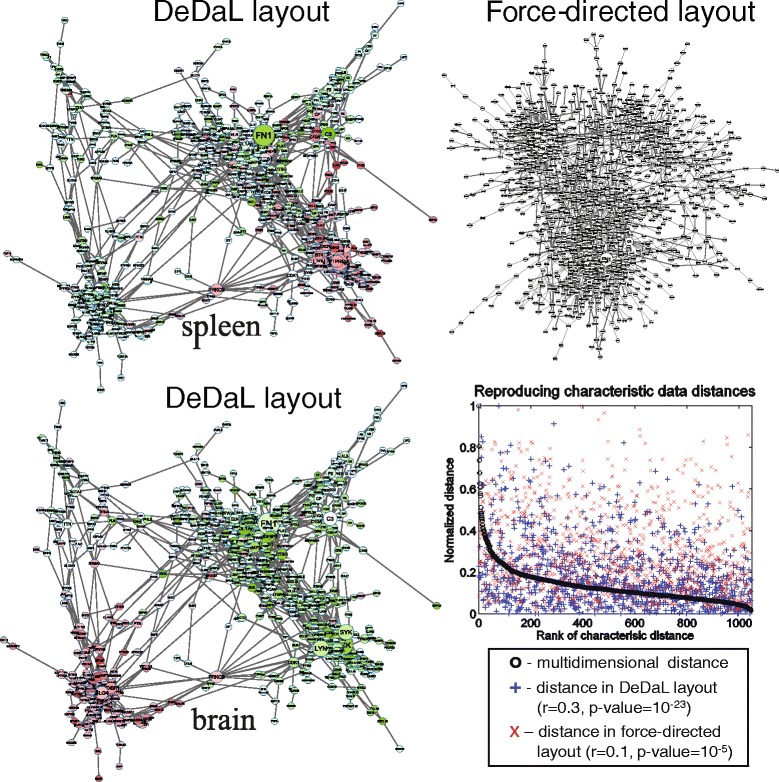
Using DeDaL for visualizing the network and RNA-Seq expression data of tissue-specific genes. RNA-Seq dataset for 27 healthy human tissues was used to defined a subnetwork of HPRD PPI database enriched in tissue-specific genes (see the text for explanations). Network smoothing followed by computation of principal manifold was applied to produce the data-driven network layout (DDL). Patterns of gene expression for two selected tissues (brain and spleen) are shown on top the constructed DDL, red color denotes higher expression, green color corresponds to lower expression. The sizes of the nodes are proportional to their connectivity degree in this network. On the left top panel application of the Force Directed layout is shown for comparison. On the left bottom panel results of quantitative comparison between multidimensional distance representation in DeDaL and Force Directed layout are shown. The most representative distances between the genes in the initial multidimensional space (see [28] for details) are ranked here from the largest to the smallest values

DeDaL was applied to this network and the whole set of tissue transcriptomes. Double-centering and network smoothing with retaining only 5 % of smallest eigenvectors was applied at the pre-processing step, and the non-linear principal manifold was computed for dimension reduction. Even without application of morphing to a structure-driven network layout, this procedure produced an insightful visualization of the network containing several clusters and connections between them (Fig. 2, left top and bottom). Mapping transcriptomes of different tissues (spleen and brain) clearly highlights different network clusters with this layout. The configuration of the clusters reflects the proximity of them in the data space of healthy tissue transcriptomes.

In order to objectively evaluate the advantage of using DeDaL for data visualization, we quantified how well the distances between the genes in the multidimensional space were reproduced on the 2D plane. We computed the correlations between gene pair-wise Euclidean distances in multidimensional space and in their configuration in a 2D network layout. We compared DeDaL with Force Directed layout frequently used to layout large networks. Since the full distance matrix contains many dependent distance values, one has to select the most representative distances for computing these correlations (pair of the most distant points, then pair of the points most distant from the first pair, etc.). Such approach was applied before to quantify the goodness of between-point distance representation after projecting them into a low-dimensional space (Quality of Distance Mapping criterion) [28]. Application of this approach in this case study showed improved Pearson correlation between the distances from 0.1 (Force Directed layout) to 0.3 (DeDaL layout), leading to increase of correlation coefficient statistical significance by 18 orders of magnitude (Fig. 2, right bottom panel). The absolute value of the correlation remains moderate because projection of multidimensional data into 2D leads to significant loss of information. Force Directed layout produces significant (though much less than DeDaL) correlation because in this case, the biological network was specifically constructed to match the variance in the dataset. The later underlines the importance of carefull biological network selection for data visualization purposes.

### Visualizing genetic interactions

Genetic interaction between two genes reflects their synergistic (negative interactions) or mutually alleviating (positive interactions) functions The strength of genetic interactions is characterized by an epistatic score which quantifies deviation from a simple multiplicative model [37]. In the global network of genetic interactions, each gene can be characterized by its epistatic profile, i.e., a vector of epistatic scores with all other genes [38]. It is shown that the genes with similar epistatic profiles tend to have similar cellular functions.

**Fig. 3:**
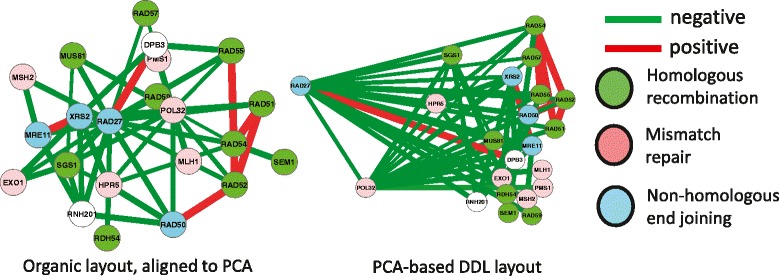
Using DeDaL for visualizing network of genetic interactions between yeast genes involved in DNA repair. Red and green edges denote positive and negative genetic interactions correspondingly. Different node colors indicate three distinct DNA repair pathways in yeast

We applied DeDaL to create a DDL layout for a group of yeast genes involved in DNA repair and replication. The genetic interactions between these genes and the epistatic profiles (computed only with respect to this group of genes) were used from [38]. The definitions of DNA repair pathways were taken from KEGG database [2]. Figure 3 shows the difference between application of the standard organic layout for this small network of genetic interactions and PCA-based DDL (computed here without applying data matrix double-centering to take into account tendencies of genes to interact with smaller or larger number of other genes). PCA-based DDL, in this case, groups the genes with respect to their epistatic profiles. Firstly, local hub genes RAD27 and POL32 have distinct position in this layout. Secondly, PCA-based DDL roughly groups the genes accordingly to the DNA repair pathway in which they are involved. For example, it shows that Non-homologous end joining (NHEJ) DNA repair pathway is closer to Homologous recombination (HR) pathway than to the Mismatch repair (MR) pathway. It also underlines that some homologous recombination genes (such as RDH54) are characterized by a different pattern of genetic interactions than the “core” HR genes RAD51, RAD52, RAD54, RAD55, RAD57.

### Visualizing attractors of a Boolean model

**Fig. 4:**
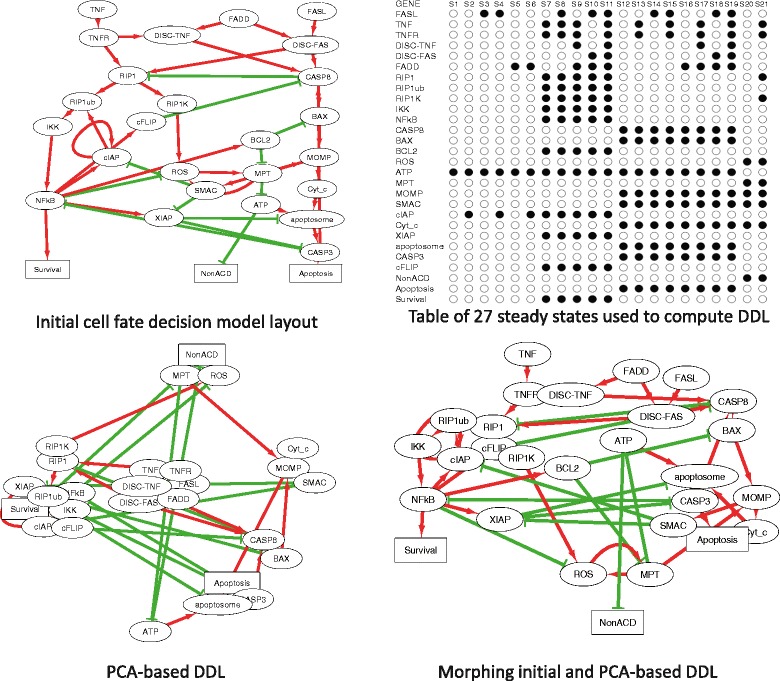
Using DeDaL for visualizing results of a Boolean model simulation. Table of computed stable states is used to group the nodes with similar states in similar conditions (shown in top right corner). In the influence diagram green edges signify inhibitory and red edges - activating relations. The model network layout is produced by applying morphing of a data-driven layout with the initial layout suggested by modeler

**Fig. 5:**
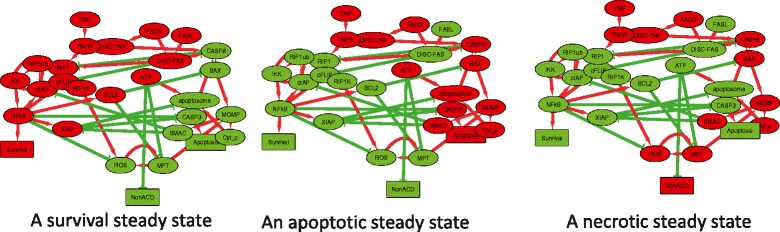
Using DeDaL for visualizing results of a Boolean model simulation. Visualization of three stable states of the model, with green and red denoting inactive (FALSE) and active (TRUE) states of the node correspondingly

In this example, we used the Boolean model of cell fate decisions between survival, apoptosis and non-apoptotic cell death (such as necrosis) published in [39], to group the nodes of the influence diagram according to their co-activation patterns in the logical stable states. The table of stable states was taken from [39] (Fig. 4, top right) and used to compute the PCA-based DDL (Fig. 4, bottom left). In this DDL, nodes in close positions have similar pattern of activation in stable states (such as RIP1 and RIP1K). We used morphing PCA-based DDL and the initial layout of the model (as it was designed in [39]) to visualize several stable states corresponding to different cell fates (Fig. 5). In this layout co-activated nodes tend to form compact groups. Therefore, DeDaL can be used to design layouts of mathematical models of biological networks, using the solutions of the model. Applying DeDaL in this analysis highlighted the functional importance of different interactions in the model and identified dynamical modules composed of variables participating in the same fate decision.

### Scalability of DeDaL for large networks

**Fig. 6:**
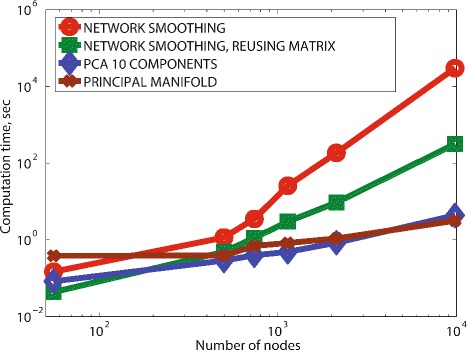
Scalability of DeDaL for large networks. The figure shows the number of seconds needed for DeDaL to compute network smoothing (red line, circles), first ten principal components (blue line, rhombes) and the principal manifold (brown line, crosses) for a set of 100 ovarian cancer transcriptomes and a series of networks with increasing number of nodes (up to 10000 nodes in the whole HPRD PPI database). Network smoothing scaling is separately shown for the case of de-novo computation of the eigenvector decomposition of the network Laplacian (red line, circles) and for the case of using the pre-computed eigenvector decomposition (green line, squares). The benchmarking was done in the command line mode of DeDaL

DeDaL scales very well with respect to computing data projections into 2D space (see Fig. 6). Even for the networks containing ten thousand nodes and more (such as the whole HPRD graph), DeDaL computes linear and non-linear data projections for few hundreds of samples in less than few tens of seconds on an ordinary laptop.

However, the network smoothing data pre-processing step implemented in DeDaL requires eigenvector decomposition of the Laplacian matrix of the network graph which scales in time as the third power of the number of nodes. While this computation remains relatively fast for relatively large networks (several minutes for a network of 2000 nodes, in our benchmark example), it drastically slows down when the size of the network grows above several thousands of nodes. In our benchmark example, eigenvector decomposition of the Laplacian of the whole HPRD PPI database required 7 hours on a regular laptop, which makes application of network smoothing data pre-processing not convenient for large networks. However, eigenvector decomposition of the Laplacian for a large graph can be done once and saved on the disk for future reuse. For example, on the DeDaL website we provide the pre-computed eigenvector decomposition for the Laplacian of the graph representing the whole HPRD database, and other decompositions for large PPI networks will be provided in the future. Using pre-computed eigenvector decomposition allows applying data network smoothing with large networks containing tens of thousands nodes in a reasonable time (few minutes).

Use of DeDaL with large networks containing tens of thousands of nodes is recommended in command line mode (see Implementation section). The computed network layout can be imported into Cytoscape environment and used for mapping high-throughput data on top of them.

## Conclusions

DeDaL Cytoscape plugin combines the classical and advanced data dimension reduction methods with the algorithms of network layouting inside Cytoscape environment. This ability can be used in a number of ways and for many applications, some of them are suggested in this paper.

The application of DeDaL is not limited to producing data-driven network layouts. More generally, DeDaL allows the application of dimension reduction of the multivariate data associated with the nodes of any Cytoscape network, optionally using the structure of the network, and exports the results for further analyses by any suitable algorithms.

## Availability and requirements

**Project name:** DeDaL: Data-Driven Network Layouting**Project home page:**http://bioinfo-out.curie.fr/projects/dedal/**Operating system(s):** Platform independent**Programming language:** Java**Other requirements:** Java 1.6 or higher, Cytoscape 3.0 or higher**License:** GNU LGPL**Any restrictions to use by non-academics:** free for any non-commercial use

## References

[1] Barillot E, Calzone L, Hupe P, Vert JP, Zinovyev A (2012). Computational Systems Biology of Cancer.

[2] Kanehisa M, Goto S, Sato Y, Furumichi M, Tanabe M (2012). Kegg for integration and interpretation of large-scale molecular data sets. Nucleic Acids Res.

[3] Croft D, O’Kelly G, Wu G, Haw R, Gillespie M, Matthews L (2011). Reactome: a database of reactions, pathways and biological processes. Nucleic Acids Res.

[4] Gehlenborg N, O’Donoghue SI, Baliga NS, Goesmann A, Hibbs MA, Kitano H (2010). Visualization of omics data for systems biology. Nat Methods.

[5] Klukas C, Schreiber F (2010). Integration of -omics data and networks for biomedical research with vanted. J Integr Bioinform.

[6] Kuperstein I, Cohen DPA, Pook S, Viara E, Calzone L, Barillot E (2013). Navicell: a web-based environment for navigation, curation and maintenance of large molecular interaction maps. BMC Syst Biol.

[7] Bonnet E, Viara E, Kuperstein I, Calzone L, Cohen DP, Barillot E (2015). Navicell web service for network-based data visualization. Nucleic Acids Res.

[8] Kuperstein I, Grieco L, Cohen D, Thieffry D, Zinovyev A, Barillot E (2015). The shortest path is not the one you know: application of biological network resources in precision oncology research. Mutagenesis.

[9] Kuperstein I, Bonnet E, Nguyen HA, Cohen D, Viara E, Grieco L (2015). Atlas of cancer signaling network: a systems biology resource for 592 integrative analysis of cancer data with google maps. Oncogenesis.

[10] Shi Z, Wang J, Zhang B (2013). Netgestalt: integrating multidimensional omics data over biological networks. Nat Methods.

[11] Ulitsky I, Shamir R (2007). Identification of functional modules using network topology and high-throughput data. BMC Syst Biol.

[12] Cline MS, Smoot M, Cerami E, Kuchinsky A, Landys N, Workman C (2007). Integration of biological networks and gene expression data using cytoscape. Nat Protoc.

[13] Alcaraz N, Friedrich T, Kötzing T, Krohmer A, Müller J, Pauling J (2012). Efficient key pathway mining: combining networks and omics data. Integr Biol (Camb).

[14] Mitra K, Carvunis AR, Ramesh SK, Ideker T (2013). Integrative approaches for finding modular structure in biological networks. Nat Rev Genet.

[15] Rapaport F, Zinovyev A, Dutreix M, Barillot E, Vert JP (2007). Classification of microarray data using gene networks. BMC Bioinformatics.

[16] Hofree M, Shen JP, Carter H, Gross A, Ideker T (2013). Network-based stratification of tumor mutations. Nat Methods.

[17] Garcia O, Saveanu C, Cline M, Fromont-Racine M, Jacquier A, Schwikowski B (2007). Golorize: a cytoscape plug-in for network visualization with gene ontology-based layout and coloring. Bioinformatics.

[18] Su G, Kuchinsky A, Morris JH, States DJ, Meng F (2010). Glay: community structure analysis of biological networks. Bioinformatics.

[19] Theocharidis A, van Dongen S, Enright AJ, Freeman TC (2009). Network visualization and analysis of gene expression data using biolayout express(3d). Nat Protoc.

[20] Pavlopoulos GA, O’Donoghue SI, Satagopam VP, Soldatos TG, Pafilis E, Schneider R (2008). Arena3d: visualization of biological networks in 3d. BMC Syst Biol.

[21] Zinovyev A, Viara E, Calzone L, Barillot E (2008). Binom: a cytoscape plugin for manipulating and analyzing biological networks. Bioinformatics.

[22] Bonnet E, Calzone L, Rovera D, Stoll G, Barillot E, Zinovyev A (2013). Practical use of binom: a biological network manager software. Methods Mol Biol.

[23] Bonnet E, Calzone L, Rovera D, Stoll G, Barillot E, Zinovyev A (2013). Binom 2.0, a cytoscape plugin for accessing and analyzing pathways using standard systems biology formats. BMC Syst Biol.

[24] Gorban A, Zinovyev A. Visualization of data by method of elastic maps and its applications in genomics, economics and sociology. IHES Preprints. 2001. (IHES/M/01/36), http://preprints.ihes.fr/M01/Resu/resu-M01-36.html.

[25] Gorban A, Zinovyev AY (2001). Method of elastic maps and its applications in data visualization and data modeling. Int J Comput Anticipatory Syst CHAOS.

[26] Gorban A, Zinovyev A (2005). Elastic principal graphs and manifolds and their practical applications. Computing.

[27] Gorban A, Kegl B, Wunsch D, Zinovyev A (2008). Principal Manifolds for Data Visualisation and Dimension Reduction, LNCSE 58.

[28] Gorban AN, Zinovyev A (2010). Principal manifolds and graphs in practice: from molecular biology to dynamical systems. Int J Neural Syst.

[29] Fung DCY, Hong SH, Koschützki D, Schreiber F, Xu K (2008). 2.5d visualisation of overlapping biological networks. J Integr Bioinform.

[30] Gorban AN, A P, Zinovyev A. Vidaexpert: user-friendly tool for nonlinear visualization and analysis of multidimensional vectorial data. Arxiv preprint(1406.5550). 2014.

[31] Gorban AN, Zinovyev A, Olivas ES, Guererro JDM, Sober MM, Benedito JRM, Lopes AJS (2009). Principal graphs and manifolds. Handbook of Research on Machine Learning Applications and Trends: Algorithms, Methods and Techniques.

[32] TCGA (2012). Comprehensive molecular portraits of human breast tumours. Nature.

[33] Peri S, Navarro JD, Kristiansen TZ, Amanchy R, Surendranath V, Muthusamy B (2004). Human protein reference database as a discovery resource for proteomics. Nucleic Acids Res.

[34] Moldovan GL, D’Andrea AD (2009). How the fanconi anemia pathway guards the genome. Annu Rev Genet.

[35] Fagerberg L, Hallström BM, Oksvold P, Kampf C, Djureinovic D, Odeberg J (2014). Analysis of the human tissue-specific expression by genome-wide integration of transcriptomics and antibody-based proteomics. Mol Cell Proteomics.

[36] Kairov U, Karpenyuk T, Ramanculov E, Zinovyev A (2012). Network analysis of gene lists for finding reproducible prognostic breast cancer gene signatures. Bioinformation.

[37] Calzone L, Barillot E, Zinovyev A (2015). Predicting genetic interactions from boolean models of biological networks. Integr Biol (Camb).

[38] Costanzo M, Baryshnikova A, Bellay J, Kim Y, Spear ED, Sevier CS (2010). The genetic landscape of a cell. Science.

[39] Calzone L, Tournier L, Fourquet S, Thieffry D, Zhivotovsky B, Barillot E (2010). Mathematical modelling of cell-fate decision in response to death receptor engagement. PLoS Comput Biol.

